# Exuberant accessory mitral valve tissue with possible true parachute mitral valve: a case report

**DOI:** 10.1186/1752-1947-6-292

**Published:** 2012-09-11

**Authors:** Aleksandra Nikolic, Zoran Joksimovic, Ljiljana Jovovic

**Affiliations:** 1 Faculty of Medicine, University of Belgrade, Serbia, Dr Subotica 8, Belgrade, Serbia; 2 Cardiovascular Institute Dedinje, Milana Tepića 1a, Belgrade, Serbia; 3 General Hospital Cuprija, Miodraga Novakovića broj 78, Cuprija, Serbia

## Abstract

**Introduction:**

A parachute mitral valve is defined as a unifocal attachment of mitral valve chordae tendineae independent of the number of papillary muscles. Data from the literature suggests that the valve can be distinguished on the basis of morphological features as either a parachute-like asymmetrical mitral valve or a true parachute mitral valve. A parachute-like asymmetrical mitral valve has two papillary muscles; one is elongated and located higher in the left ventricle. A true parachute mitral valve has a single papillary muscle that receives all chordae, as was present in our patient. Patients with parachute mitral valves during childhood have multilevel left-side heart obstructions, with poor outcomes without operative treatment. The finding of a parachute mitral valve in an adult patient is extremely rare, especially as an isolated lesion. In adults, the unifocal attachment of the chordae results in a slightly restricted valve opening and, more frequently, valvular regurgitation.

**Case presentation:**

A 40-year-old Caucasian female patient was admitted to a primary care physician due to her recent symptoms of heart palpitation and chest discomfort on effort. Transthoracic echocardiography showed chordae tendineae which were elongated and formed an unusual net shape penetrating into left ventricle cavity. The parasternal short axis view of her left ventricle showed a single papillary muscle positioned on one side in the posteromedial commissure receiving all chordae. Her mitral valve orifice was slightly eccentric and the chordae were converting into a single papillary muscle. Mitral regurgitation was present and it was graded as moderate to severe. Her left atrium was enlarged. There were no signs of mitral stenosis or a subvalvular ring. She did not have a bicuspid aortic valve or coarctation of the ascending aorta. The dimensions and systolic function of her left ventricle were normal. Our patient had a normal body habitus, without signs of heart failure. Her functional status was graded as class I according to the New York Heart Association grading.

**Conclusions:**

A recently published review found that, in the last several decades, there have been only nine adult patients with parachute mitral valve disease reported, of which five had the same morphological characteristics as our patient. This case presentation should encourage doctors, especially those involved in echocardiography, to contribute their own experience, knowledge and research in parachute mitral valve disease to enrich statistical and epidemiologic databases and aid clinicians in getting acquainted with this rare disease.

## Introduction

The parachute mitral valve (PMV) is defined as a unifocal attachment of mitral valve chordae independent of a number of papillary muscles. In 1963, Shone *et al*. originally described the developmental complex that included PMV, supravalvular ring of the left atrium, subaortic stenosis and coarctation of the aorta, and reported that the degree of mitral valve involvement appeared to be the main factor determining the outcome in referring patients [1]. Shone et al. showed that development of the anterolateral and posteromedial papillary muscles was disrupted between the fifth and nineteenth week of gestation, thereby forcing the embryonic predecessors of the papillary muscles to condense into a single muscle and the only effective communication between the left atrium and the left ventricle through the interchordal spaces [1]. Oosthoek et al. suggested that the valve can be distinguished on the basis of morphological features into either parachute-like asymmetrical mitral valves or a true PMV. Parachute-like asymmetrical mitral valves have two papillary muscles, with one elongated and located higher in the left ventricle, with its tip reaching to the annulus and attached to the base of both muscles and the lateral side to the left ventricular wall. True PMV have a single papillary muscle that receives all chordae. A PMV with a single papillary muscle is rarer than the asymmetrical parachute-like mitral valve [2,3]. Most patients are diagnosed in their childhood, and patients of this age with multilevel left-side heart obstructions and mitral valve involvement, which is severely stenotic, usually have poor outcomes. The management of congenital mitral valve stenosis and associated lesions in the pediatric population is a major challenge, although several authors have reported excellent results after surgery [3-6].

## Case presentation

In the middle of 2008, a 40-year-old Caucasian female patient was admitted to a primary care physician due to her recent symptoms of heart palpitation and chest discomfort on effort. Our patient had successfully delivered her second child a few months prior to presentation. She underwent detailed physical observation in a regional medical center and it was recommended that she had a transthoracic echocardiography examination. She was referred to our hospital in January 2009 for further evaluation.

Our patient had a normal body habitus. An electrocardiogram showed sinus tachycardia. Her heart rate was around 90 beats/min. Holter monitoring showed sinus rhythm, heart rate between 49 and 149 beats/min, 9 supraventricular ectopic beats and 13 ventricular ectopic beats. Heart auscultation revealed a pansystolic murmur, graded 4/6, audible over the apex. She had no coronary artery risk factors. She was graded as New York Heart Association functional class I. Laboratory data (including thyroid hormones) were normal and other findings were regular.

**Figure 1:**
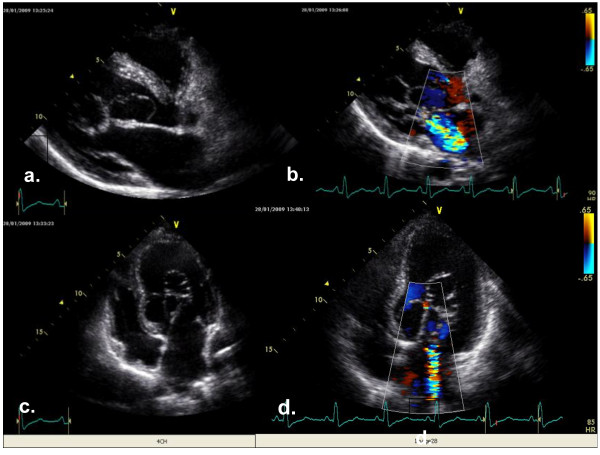
**Parasternal long axis and four-chamber view of an unusual presentation of a mitral valve. (a)** Parasternal long axis view shows chordae meshwork into the left ventricle cavity. **(b)** Parasternal long axis view of color Doppler shows moderate to severe mitral regurgitation. **(c)** Four-chamber view shows elongated chordae that were predominantly oriented to the septum. **(d)** Four-chamber view of color Doppler confirmed the presence of moderate to severe mitral regurgitation.

**Figure 2:**
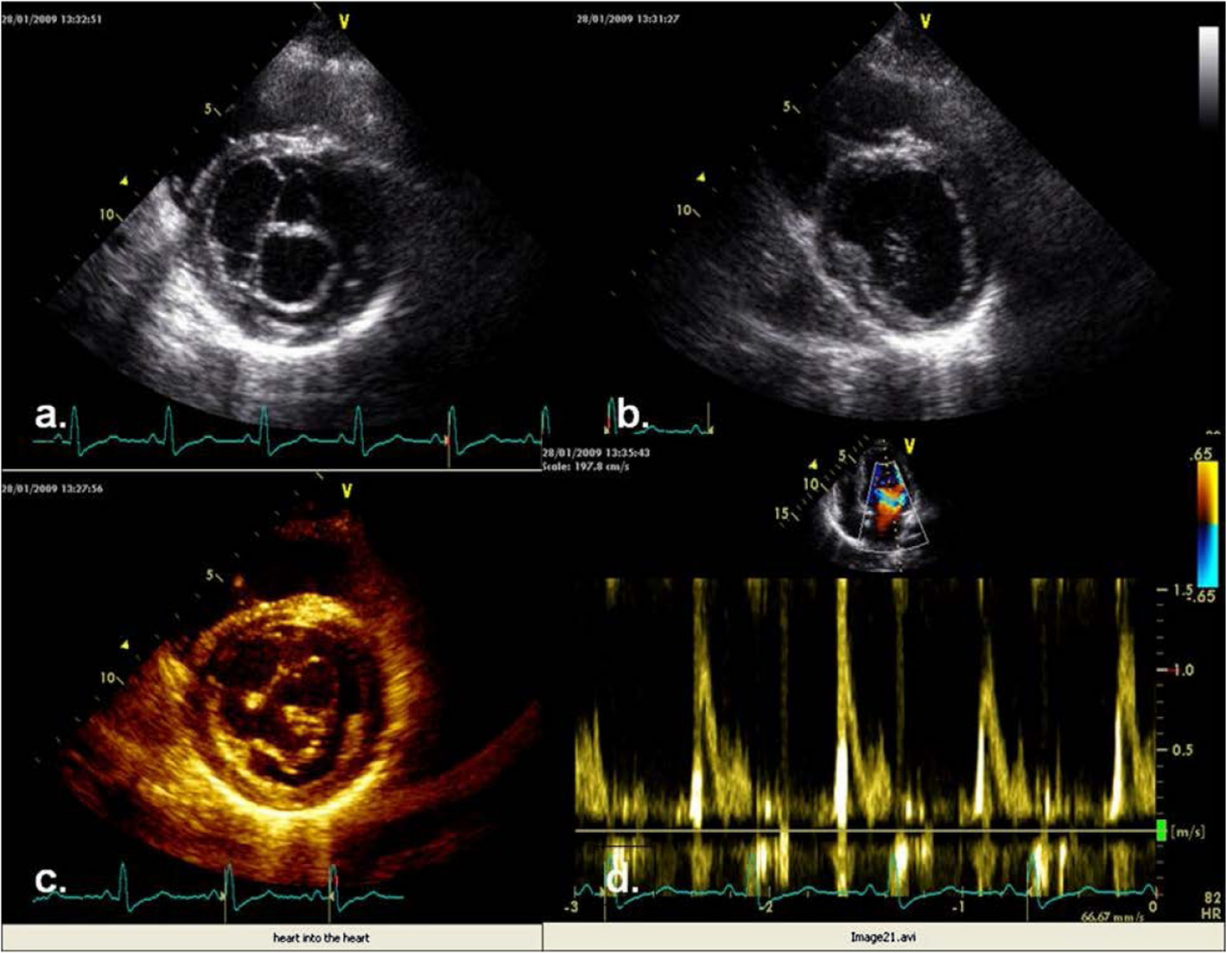
**Parasternal long axis view revealed chord meshwork, with normal pulsed-wave Doppler flow. (a)** Parasternal long axis view on the level of the mitral valve shows eccentric mitral valve orifice, predominantly oriented to the posteromedial commissure. **(b)** Parasternal long axis view on the level of papillary muscle shows a single posteromedial muscle. **(c)** ‘Heart into the heart’ orientation of the chordae. **(d)** Doppler transmitral flow shows normal relaxation of the left ventricle.

A transthoracic echocardiography examination was performed using a GE Healthcare Vivid 7 Dimension Cardiovascular Ultrasound System (GE Healthcare, Milwaukee, WI, USA). Her left atrium was enlarged (44mm) and the parasternal long axis showed chordae tendineae that were elongated and formed an unusual net shape, penetrating into her left ventricular cavity (Figure 1a,c) (Additional file 1: Movie 1 and Additional file 2: Movie 2). The parasternal short axis of her left ventricle showed a single papillary muscle that was positioned on one side in the posteromedial commissure (Figure 2a,b). The mitral valve orifice was slightly eccentric and all chordae were converting into a single papillary muscle (Figure 2c). Transmitral flow showed a normal pattern (Figure 2d). Mitral regurgitation was present and was graded as moderate to severe (Figure 1b,d). The dimensions of her left ventricle cavity were normal (end-diastolic dimension, 56mm; end-systolic dimension, 38mm), and revealed normal segmental wall motion with preserved ejection fraction. There was no obstruction of the left ventricular outflow tract. Her aortic valve had three leaflets, with no regurgitation or stenosis. The blood-flow velocity through her aorta was normal. There was no coarctation of her aorta. The right side of her heart was normal. Tricuspidal regurgitation was mild without elevated right heart pressure. Her pericardium was normal.

In accordance with the guidelines for valvular heart disease, and given that our patient was oligosymptomatic with left ventricular size and function within the normal range, the recommendation was to follow monitoring our patient until her operation [7]. After initiation of therapy with beta blockers, our patient’s symptoms have completely reduced. Our patient has a twin sister, who was without any symptoms and whose physical status and echocardiography findings were normal.

## Conclusions

The finding of a PMV in an adult patient is extremely rare, especially as an isolated lesion. In adults, the unifocal attachment of the chordae leads to restricted valve opening and, more frequently, valvular regurgitation. Case presentations with individual experience should encourage doctors, especially those involved in echocardiography, to contribute their experience, knowledge and research to enrich statistical and epidemiologic databases and aid clinicians in getting acquainted with this rare disease.

## Consent

Written informed consent was obtained from the patient for publication of this case report and accompanying images. A copy of the written consent is available for review by the Editor-in-Chief of this journal.

## Competing interests

The authors declare that they have no competing interests.

## Authors’ contributions

AN analyzed and interpreted the patient data regarding the PMV and was a major contributor in writing the manuscript. ZJ revealed the heart disorder in our patient, treated our patient medically and continues to perform medical examinations and transthoracic echocardiography follow-ups. LJ was the supervisor in proving the diagnosis of PMV and contributed to comparing our patient’s echocardiographic data with those cases described in the literature. All authors read and approved the final manuscript.

## Supplementary Material

Additional file 1 **Movie 1.** Parasternal long axis view shows chordae meshwork into the left ventricle cavity. Click here for file

Additional file 2 **Movie 2.** Four-chamber view shows elongated chordae that were predominantly oriented to the septum. Click here for file
